# Molecular investigation of *Cryptosporidium* in farmed chickens in Hubei Province, China, identifies ‘zoonotic’ subtypes of *C. meleagridis*

**DOI:** 10.1186/s13071-018-3056-5

**Published:** 2018-08-29

**Authors:** Cong Liao, Tao Wang, Anson V. Koehler, Yingying Fan, Min Hu, Robin B. Gasser

**Affiliations:** 1 State Key Laboratory of Agricultural Microbiology, College of Veterinary Medicine, Huazhong Agricultural University, Wuhan, 430070 Hubei China; 2 Department of Veterinary Biosciences, Melbourne Veterinary School, The University of Melbourne, Parkville, Victoria Australia

**Keywords:** *Cryptosporidium*, Bird, Human, Zoonosis, China, PCR-based sequencing, Phylogenetic analyses

## Abstract

**Background:**

*Cryptosporidium* is a key genus of parasitic protists that infect humans and other vertebrates (mammals and birds). Birds are typically infected with *C. avium*, *C. baileyi*, *C. galli* and/or *C. meleagridis*, the latter of which is recognised as being zoonotic. Stimulated by the previous finding of *C. meleagridis* subtypes IIIbA21G1R1, IIIbA22G1R1 and IIIbA26G1R1 in diarrhoeic children in Wuhan city and environs in Hubei Province, China, we performed a molecular epidemiological survey to explore whether these or similar subtypes might occur in farmed chickens in this province.

**Methods:**

PCR-coupled sequencing analyses of regions in the small subunit (*SSU*) of the nuclear ribosomal RNA and 60 kDa glycoprotein (*gp60*) genes were utilised to characterise *Cryptosporidium* in faecal samples from chickens (*n* = 471) from 14 farms from six distinct regions in Hubei Province.

**Results:**

*Cryptosporidium baileyi* (33/471; 7.0%) and *C. meleagridis* (15/471; 3.2%) were identified in chickens on eight farms in five of the six distinct geographical regions. No significant age-associated difference in the prevalence of *C. baileyi* was evident, whereas the prevalence of *C. meleagridis* was significantly higher in younger (≤ 4 months) than in older chickens (> 4 months). For *C. meleagridis*, two subtype families, IIIb and IIIe, were defined; some of the subtypes (i.e. IIIbA26G1R1b and IIIbA22G1R1c) characterised here matched those identified previously in diarrhoeic children in Wuhan.

**Conclusions:**

This is the first molecular study reporting the genetic identity and prevalence of *C. baileyi* and *C. meleagridis* in chickens in Hubei. The findings suggest that *C. meleagridis* subtypes IIIbA26G1R1b and IIIbA22G1R1c are cross-transmissible between chickens and humans, raising awareness about the significance of birds as potential reservoirs of zoonotic variants of *Cryptosporidium.* Future studies might focus on investigating the prevalence of ‘zoonotic’ subtypes of *Cryptosporidium meleagridis* in various species of wild and domesticated birds, and on comparing them with those found in humans in China and other countries.

**Electronic supplementary material:**

The online version of this article (10.1186/s13071-018-3056-5) contains supplementary material, which is available to authorized users.

## Background

*Cryptosporidium* is a socioeconomically significant genus of parasitic protists that infect humans and other vertebrates worldwide. Species within this genus are transmitted *via* the faecal-oral route, often through direct contact with infected people or animals, food and/or water [1–4], resulting in gastrointestinal disease in mammals, or respiratory and gastrointestinal infections in birds. Clinical signs linked to human cryptosporidiosis include diarrhoea, dehydration, vomiting, wasting and/or weight loss [5, 6], although subclinical infections can occur [7, 8]. Cryptosporidiosis can resolve as immunity develops to clear an infection [9], but chronic disease can develop in at-risk individuals, including young children and people with immuno-suppression or deficiency [10]. In the absence of readily accessible, effective chemotherapeutics and immunoprophylactics [11, 12], chronic cryptosporidiosis can cause death, particularly in patients seriously affected by HIV/AIDS [13, 14].

Molecular epidemiological investigations utilising PCR-coupled sequencing of particular genetic markers, such as those in the small subunit (*SSU*) of nuclear ribosomal RNA and the 60 kDa glycoprotein (*gp60*) genes, have shown that human cryptosporidiosis is predominantly caused by *Cryptosporidium hominis* or *C. parvum* infection [7, 15], although symptomatic or non-symptomatic infections have been linked to species (*n* ≥ 17) including *C. felis*, *C. canis* and *C. meleagridis* or various genotypes (*n* ≥ 4) [15–17]. Cryptosporidiosis cases have been associated with human-to-human (anthroponotic) transmission for *C. hominis* and *C. parvum*, and animal-to-human (zoonotic) transmission for taxa including *C. parvum*, *C. meleagridis*, *C. canis* and *C. felis* [7, 15, 16, 18–22].

In a recent molecular epidemiological survey [23], we were surprised to identify *C. meleagridis* subtype IIIb (specifically IIIbA21G1R1, IIIbA22G1R1 and IIIbA26G1R1) in 2% of 500 children with a history of diarrhoea in Wuhan and environs in Hubei Province, China, although this prevalence was similar to some previous studies of children in other parts of China [24, 25]. *Cryptosporidium meleagridis* is primarily a pathogen of birds (e.g. chickens, cockatiels, parrots, pigeons and turkeys) [4, 16, 26–30], and this species has been recorded mainly in immuno-compromised persons and in children [11, 31–33], with a potential to lead to chronic cryptosporidiosis [34]. The source of *C. meleagridis* infection in humans was unclear in most published reports, and the assumption has been that birds can be significant reservoirs of this protist, although anthroponotic transmission might also occur [15–17, 30]. A study by Chappell et al. [34] established that healthy adults could be infected by *C. meleagridis* oocysts, presenting with gastrointestinal symptoms including diarrhoea. Based on the findings of our recent investigation [23], we emphasised the need to explore the presence and prevalence of *C. meleagridis* in domestic and wild birds*.* In the present study, we take a first step by investigating *Cryptosporidium* in intensively farmed chickens in Hubei Province using molecular tools.

## Methods

**Fig. 1:**
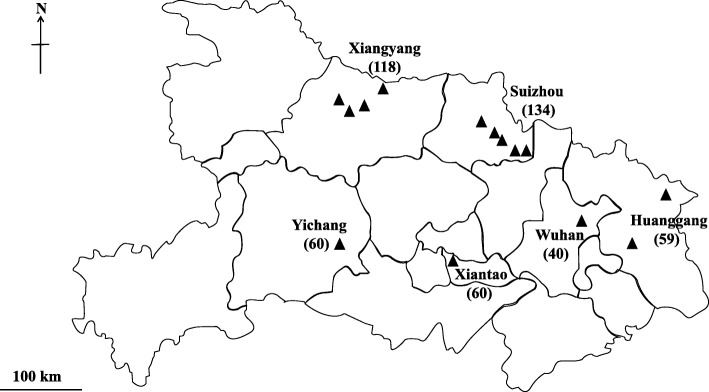
Geographical locations of the 14 farms in Hubei Province, China, from which faecal samples (numbers in parentheses) were collected from chickens

Between July and November 2017, 471 fresh faecal samples were collected from chickens of different age groups from 14 medium- to large-sized farms (each with 2000–25,000 broilers or layers on average) in six distinct geographical regions (Huanggang, Suizhou, Wuhan, Xiantao, Xiangyang and Yichang) in Hubei Province, China (Fig. 1; Table 1). For broilers, 14 to 60 samples were collected per farm (with each sample representing 4–5 faecal deposits randomly collected from flocks of 30–100 chickens each). For layers, 19 to 60 samples were collected per farm (with each sample representing a cage of 5–7 chickens). Genomic DNAs were extracted from individual faecal samples using the PowerSoil DNA isolation kit (MoBio, Carlsbad, USA) and frozen at -20 °C.

**Table 1:** Occurrence of *Cryptosporidium meleagridis* and *Cryptosporidium baileyi* in faecal samples from chickens from broiler or layer farms in six distinct locations in Hubei Province, China (cf. Fig. 1)

Location	Farm	No. of samples tested	No. of samples test-positive for *Cryptosporidium* spp. (%)	*Cryptosporidium* species	
				*C. baileyi* (%)	*C. meleagridis* (%)
Huanggang	Farm A (layers)	30	4 (13.3)	0	4 (13.3)
	Farm B (layers)	29	4 (13.8)	4 (13.8)	0
Wuhan	Farm C (layers)	40	7 (17.5)	1 (2.5)	6 (15.0)
Suizhou	Farm D (layers)	40	2 (5.0)	2 (5.0)	0
	Farm E (layers)	19	0	0	0
	Farm F (broilers)	14	0	0	0
	Farm G (layers)	21	7 (33.3)	7 (33.3)	0
	Farm H (layers)	40	5 (12.5)	5 (12.5)	0
Yichang	Farm I (broilers)	60	0	0	0
Xiangyang	Farm J (layers)	51	8 (15.7)	6 (11.8)	2 (3.9)
	Farm K (layers)	20	0	0	0
	Farm L (broilers)	29	0	0	0
	Farm M (broilers)	18	0	0	0
Xiantao	Farm N (layers)	60	11 (18.3)	8 (13.3)	3 (5.0)
Total		471	48 (10.2)	33 (7.0)	15 (3.2)

Individual DNAs were subjected to nested PCR-based amplification and sequencing of regions of the small subunit of the nuclear ribosomal RNA gene (designated p*SSU*; ~240 bp) [35] and the 60 kDa glycoprotein gene (designated p*gp60*; ~900 bp) of *Cryptosporidium* for classification to the genotype and subtype levels [33]. PCR was conducted in 50 μl containing 50 mM KCl and 10 mM Tris-HCl (pH 8.4; Promega, Madison, USA), 3.0 mM of MgCl_2_, 200 μM of each deoxynucleotide triphosphate, 50 pmol of each primer and 1 U of Mango*Taq* DNA polymerase (Bioline, London, UK). Known test-positive, test-negative and no-template (including ‘carry-over’) controls were included in each step of each set of PCRs. PCR products were resolved on 1.5% agarose gels, stained with ethidium bromide prior to sequencing. Then, aliquots (5 μl) of individual amplicons (undigested) were treated with the enzyme *Exo* I and a thermosensitive alkaline phosphatase (FastAP, Thermo Fisher, Carlsbad, USA), according to the manufacturer's instructions, and subjected to automated sequencing (BigDye Terminator v.3.1 chemistry, Applied Biosystems, Foster City, USA) employing the same primers (separately) as used in PCR.

Sequences were aligned using the program MAFFT [36], and alignments manually adjusted employing the program Mesquite v.3.10 [37]. Sequences were then compared with sequence data available *via* GenBank (NCBI) using BLASTn (Additional file 1: Table S1). Phylogenetic analysis of p*SSU* or p*gp60* sequence data (including selected reference sequences; Additional file 1: Table S1) was conducted by Bayesian inference (BI) using Monte Carlo Markov Chain (MCMC) analysis in MrBayes v.3.2.6 [38]. The likelihood parameters set for BI analysis of p*gp60* data were based on the Akaike Information Criteria test [39] in jModeltest v.2.1.7. The number of substitutions (Nst) was set at 6, with an invariant gamma-distribution. Posterior probability (pp) values were calculated by running 2,000,000 generations with four simultaneous tree-building chains. Trees were saved every 100th generation. At the end of each run, the standard deviation of split frequencies was < 0.01, and the potential scale reduction factor approached one. A 50% majority rule consensus tree for each analysis was constructed based on the final 75% of trees generated by BI. Analyses were run three times to ensure convergence and insensitivity to priors. The outgroups used in the phylogenetic analyses of p*SSU* and p*gp60* sequence data sets were *C. molnari* (GenBank: HM243547) and *C. meleagridis* subtype IIId (GenBank: DQ067570.1), respectively. The Chi-square test was performed using SPSS Statistics 24 software (IBM, New York, USA).

## Results

All 471 individual faecal samples from chickens were analysed molecularly for the presence of *Cryptosporidium* species, genotypes and subtypes. The p*SSU* amplicons were generated from 48 of the 471 DNA samples, equating to an overall prevalence of *Cryptosporidium* of 10.2% (Table 1), with prevalence values ranging from 5.0% to 18.3% on eight of 14 farms from the five of the six geographical regions (Table 1). *Cryptosporidium* was detected exclusively in layer chickens, but not in broilers (Table 1).

*Cryptosporidium* was detected in both age groups (Table 2), and young chickens (≤ 4 months) tended to have a higher infection rate (15.1%) than the chickens of > 4 months (11.7%). *Cryptosporidium baileyi* and *C. meleagridis* were detected in birds of both age groups. The prevalence of *C. baileyi* seemed higher in older chickens (> 4 months), although there was no statistical difference between age groups (*χ*^2^ = 0.75, *df* = 1, *P* = 0.387). In contrast, the prevalence of *C. meleagridis* was higher in younger chickens (≤ 4 months) (*χ*^2^ = 7.8, *df* = 1, *P* = 0.005) (Table 2).

**Table 2:** Age groups of chickens in which *Cryptosporidium* species were detected using PCR-based tools (cf. Table 1)

Age group	Test-positive/total no. tested (%) for *Cryptosporidium*	Test-positive/total no. tested (%) for *Cryptosporidium baileyi*	Test-positive/total no. tested (%) for *Cryptosporidium meleagridis*
≤ 4 months	31/205 (15.1)	17/205 (8.3)	14/205 (6.8)
> 4 months	17/145 (11.7)	16/145 (11.0)	1/145 (0.7)
Total	48/350 (13.7)	33/350 (9.4)	15/350 (4.3)

**Fig. 2:**
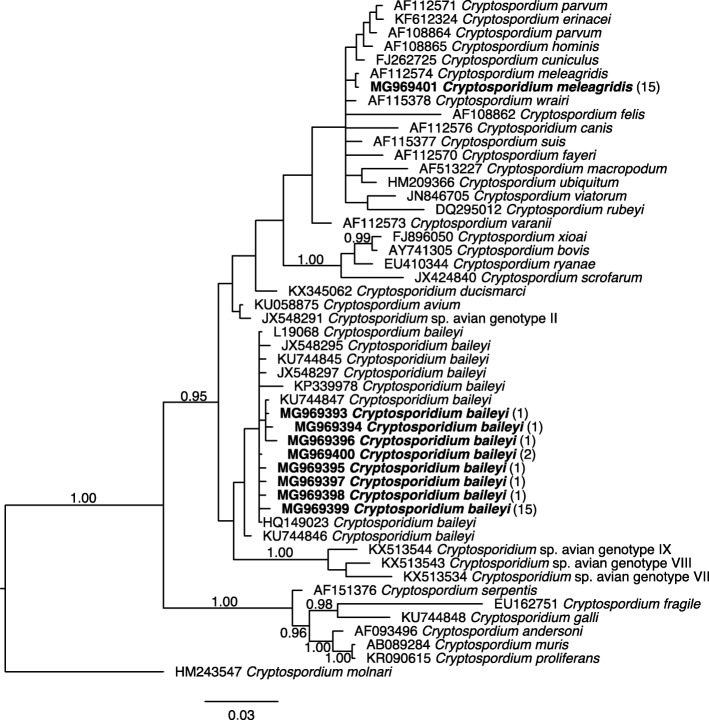
Relationships among *Cryptosporidium* taxa inferred from the phylogenetic analysis of sequences from a portion of the small subunit of the nuclear ribosomal RNA gene (p*SSU*) by Bayesian inference (BI). Posterior probabilities of > 0.95 are indicated at all major nodes. Bold-type indicates *Cryptosporidium* species or genotypes characterised from faecal DNA samples in this study. The GenBank accession number precedes the species designation; the number of samples of a particular species/genotype is indicated in parentheses. The scale-bar represents the number of substitutions per site. *Cryptosporidium molnari* was used as an outgroup

The identification of *Cryptosporidium* species and genotypes was achieved through the sequencing of p*SSU* amplicons (*n* = 48). This analysis revealed *C. baileyi* in 68.8% and *C. meleagridis* in 31.2% of the 48 samples; no mixed-species infections were detected. Nine distinct p*SSU* sequences that represented all 48 samples and both *Cryptosporidium* species were deposited under GenBank accession numbers MG969393-MG969401, and the relationships of these sequences with selected reference sequences from GenBank (Additional file 1: Table S1) were established through a phylogenetic analysis (Fig. 2). Specifically, eight of these sequences (GenBank: MG969393-MG969400) clustered with known p*SSU* reference sequences for *C. baileyi*, and the other one (GenBank: MG969401) clustered with a representative sequence for *C. meleagridis*, on a branch with ‘zoonotic’ species including *C. felis*, *C. hominis* and *C. parvum* (see Fig. 2).

**Fig. 3:**
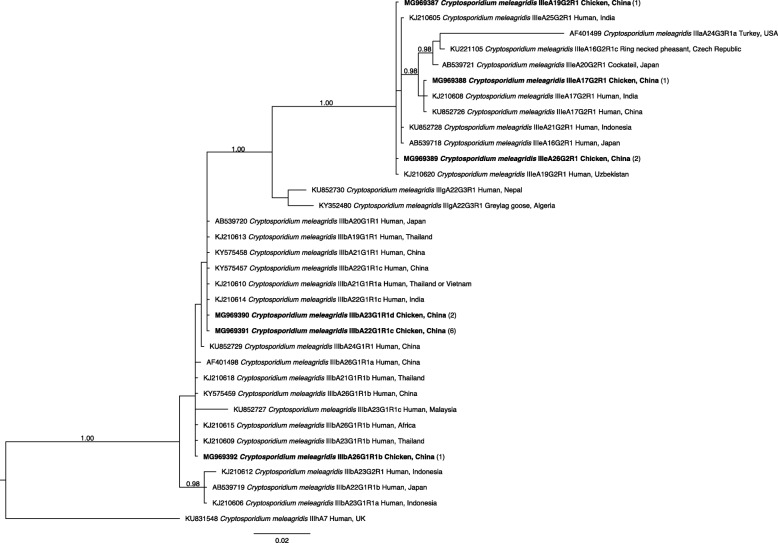
Relationships among *Cryptosporidium meleagridis* subtypes inferred from the phylogenetic analysis of sequences from a portion of the nuclear 60-kDa glycoprotein gene (p*gp60*) by Bayesian inference (BI). Posterior probabilities of > 0.95 are indicated at all major nodes. Bold-type indicates subtypes characterised from faecal DNA samples tested in this study. The GenBank accession number precedes the species designation; the number of samples of a particular species/genotype is indicated in parentheses. The scale-bar represents the number of substitutions per site. Subtype IIIhA7 was used as an outgroup

Subtyping was achieved through an analysis of sequence data derived from *gp60* amplicons obtained (*n* = 13; deposited under GenBank: MG969387-MG969392). Sequence alignment and phylogenetic analysis of the sequences revealed six new subtypes of *C. meleagridis* in chickens. This analysis defined two subtype families (IIIb and IIIe; Fig. 3); the commonest subtype family IIIb [IIIbA22G1R1c (*n* = 6); IIIbA23G1R1d (*n* = 2) and IIIbA26G1R1b (*n* = 1)] was identified for nine samples, and subtype family IIIe [IIIeA17G2R1 (*n* = 1), IIIeA19G2R1 (*n* = 1) and IIIeA26G2R1 (*n* = 2)] for four samples. The phylogenetic analysis showed that nine sequences representing *C. meleagridis* subtype IIIb (GenBank: MG969390-MG969392) clustered with p*gp60* sequences (GenBank: KY575457-KY575459) derived from samples from diarrhoeic children from Wuhan [23]. The p*gp60* gene sequences representing *C. meleagridis* subtype IIIb from chickens (GenBank: MG969390-MG969392) showed high sequence similarity (92.7–100%) with those from humans (GenBank: KY575457-KY575459), being associated with subtypes IIIbA22G1R1c, IIIbA23G1R1d and IIIbA26G1R1b.

## Discussion

The present molecular investigation reports, for the first time, the presence and prevalence of *Cryptosporidium* in chickens on commercial farms in Hubei Province, although previous studies have recorded *Cryptosporidium* in chickens in Henan [40] and Zhejiang [41]. Here, both *C. baileyi* and *C. meleagridis* were identified using PCR-based tools. The overall prevalence of *Cryptosporidium* in chickens was ~10%, which is consistent with percentages recorded previously in China (8.9%, Henan; 9.9%, Zhejiang) [40, 41] and Syria (9.9%) [42], higher than Jordan (4.8%) [43] and Tunisia (4.5%) [44], and lower than Brazil (12.6%) [45]. *Cryptosporidium* infection has been recorded mainly in broiler chickens in countries including China, Algeria, Germany, Iran, Syria and Tunisia [26, 41, 42, 44, 46, 47]. Published studies indicate that young birds are more frequently infected with *Cryptosporidium* than adults [28, 40, 46], but more work is needed to confirm such an age-related association.

*Cryptosporidium baileyi* was detected in most (69%) of the 48 test-positive chicken faecal samples*. Cryptosporidium baileyi* was originally isolated from commercial broiler chickens [48], has been recorded in a broad range of avian hosts and is considered to be a dominant species in birds, although other taxa including *C. avium*, *C. galli* and/or *C. meleagridis* occur, of which only the latter species is recognised as zoonotic [4, 30, 49]. In China, *C. baileyi* has been reported in farmed and wild birds, including chickens, quails, ostriches, Pekin ducks, domestic pigeons as well as some pet birds (e.g. rufous turtle dove, zebra finch, red-billed leiothrix, black-billed magpie and white Java sparrow) [27, 40, 50–54]. Previous reports indicate that *C. baileyi* causes reduced weight gain in broilers and decreased egg production in layer chickens, often in the absence of obvious clinical signs [55, 56]. We propose that *C. baileyi* may be a species of economic and/or clinical importance, given its relatively broad distribution on most farms studied here, with the exception of Yichang. Although *C. baileyi* has been recorded in an immunodeficient human patient [57], the zoonotic potential of this species is questionable.

*Cryptosporidium meleagridis* was detected in almost one third (31%) of the 48 test-positive chicken faecal samples. Although commonly identified in avian hosts, *C. meleagridis* has been found in humans in a number of countries, including Australia, South Africa, China, France, India, Indonesia, Japan, Jordan, Kenya, Nigeria, Peru, Poland, Portugal, Spain, Sweden, Thailand, Tunisia, the United Kingdom and Uzbekistan [23, 31, 33, 58–70]. Both immunocompetent and immunocompromised humans can be infected/affected, indicating the public health significance of this species. In China, *C. meleagridis* has been recorded previously in paediatric patients, HIV-positive individuals in Shanghai and Henan provinces, respectively, and animal contact has been discussed as a significant risk factor [24, 68]; moreover, there is clear molecular evidence of *C. meleagridis* subtypes being shared by humans and birds. For instance, in Sweden, an outbreak of *C. meleagridis* infection/cryptosporidiosis in people was confirmed by PCR-based sequence analyses of *SSU* and heat shock protein 70 (*hsp70*) gene regions; *C. meleagridis* genotype I (GenBank: AF12574) defined in humans was the same as found in chickens [21]. In Peru, two subtypes of *C. meleagridis* (MLG1 and MLG8) characterised from HIV/AIDS patients were shared by birds (chicken, pigeon or duck) in the same location (Lima, Peru) through genetic analyses of *gp60* and mini-satellites [71]. Importantly, p*gp60* subtypes of *C. meleagridis* (IIIbA22G1R1c and IIIbA26G1R1b) characterised from diarrhoeic children in Wuhan [23] match those identified in chickens in the present study. These findings suggest that, in Wuhan and environs, chickens may contribute to the transmission of *C. meleagridis* to humans. Whether wild or other domesticated (e.g. pet) birds might be involved in such transmission requires detailed investigation.

In the present study of chickens, we defined subtype families IIIb and IIIe of *C. meleagridis* based on p*gp60* sequence data. Both of these families have been recorded previously, mainly in humans and occasionally in birds (cockatiel and turkey) and cattle in countries including China, Indonesia, Japan, Kenya, Peru, Thailand and the USA [33]. Within these families, subtypes IIIbA22G1R1c, IIIbA26G1R1b, IIIeA17G2R1, IIIeA19G2R1 and IIIeA26G2R1 have been recorded in humans [33, 68, 72], and were identified here in chickens in Hubei Province. Subtype IIIbA22G1R1c was the predominant subtype in this and our previous study of children [23]. This subtype has been detected in people with travel-acquired infections. For example, subtype IIIbA22G1R1c, detected in some people from Sweden, was linked to travel to India or Thailand [21, 33]. The same subtype had been recorded in an English patient proposed to have become infected in India (sample analysed in the UK) [33, 69]. This information highlights the potential of this pathogen and various IIIb subtypes to be spread worldwide through human travel. In this context, subtype IIIbA23G1R1d was found here, for the first time, in chickens. However, subtype IIIbA23G1R1 had been recorded in quail in Brazil, and a human in Peru [71, 73], and IIIbA23G1R1a, IIIbA23G1R1b and IIIbA23G1R1c in people with histories of travel to Thailand, Indonesia and Malaysia, respectively [21, 33, 74]. We propose that subtype IIIbA23G1R1d might be transmissible from poultry to humans, but this needs to be explored in detail.

## Conclusions

This is the first molecular study reporting the genetic identity and prevalence of *C. baileyi* and *C. meleagridis* in chickens in Hubei. The findings suggest that *C. meleagridis* subtypes IIIbA26G1R1b and IIIbA22G1R1c are cross-transmissible between chickens and humans, raising awareness about the significance of birds as potential reservoirs of zoonotic variants of *Cryptosporidium*. Taken together, this information emphasises the need for epidemiological studies of *Cryptosporidium*, particularly *C. meleagridis*, in humans and in birds using accurate diagnostic and analytical tools utilising multiple informative genetic loci. Such investigations should be focused on assessing the transmission patterns and dynamics of cryptosporidiosis.

## Additional file

Additional file 1: **Table S1.** Summary of information on the reference sequences from the GenBank database used in the present study. (XLSX 21 kb)

## Abbreviations

p*SSU*: a portion of the small subunit of nuclear ribosomal RNA gene
p*gp60*: a portion of the 60 kDa glycoprotein gene
